# Professor Elias Wildman

**Published:** 1876-09

**Authors:** 


### OBITUARY.

---

Professor Elias Wildman, so widely known to the dental profession from his minute investigations of Vulcanized Rubber, the results of which are published in a work entitled "Instructions in the Manipulation of Hard Rubbes or Vulcanite for Dental Purposes," died suddenly on the 25th of July last, in the sixty-sixth year of his age. Since 1862 Professor Wildman held the chair of Mechanical Dentistry in the Pennsylvania Dental College, and since 1871 has been Dean of that institution. He was a man of the greatest energy and perseverance, and what he undertook was well performed in every respect.

His gum enamel for porcelain teeth, the result of experiments commenced soon after he entered the dental profession in 1836, is still considered unsurpassed. Prof. Wildman practiced medicine prior to entering the dental profession, and while connected with the Pennsylvania Dental College was regarded as a most successful teacher and one thoroughly acquainted with all the principles of the branch of the science he taught.

His loss will be severely felt and deeply deplored.

